# MicroRNAs won the Nobel Prize. Now, can extracellular vesicles help them become drugs?

**DOI:** 10.1016/j.vesic.2025.100080

**Published:** 2025-04-21

**Authors:** Vanessa YiRan Li, Nicole Rosas, Sharon Fleischer, Gordana Vunjak-Novakovic, Ke Cheng

**Affiliations:** a Department of Biomedical Engineering, Columbia University, New York, NY, USA; b Department of Medicine, Columbia University, New York, NY, USA; c College of Dental Medicine, Columbia University, New York, NY, USA; d Herbert Irving Comprehensive Cancer Center, Columbia University, New York, NY, USA

**Keywords:** Extracellular vesicles, MicroRNAs, Nobel Prize in medicine, Therapeutics, Clinical translation, Commercialization

## Abstract

The 2024 Nobel Prize recognized microRNAs (miRNAs) as transformative regulators of gene expression. However, their clinical potential has been constrained by instability and delivery challenges. Extracellular vesicles (EVs), as natural carriers of miRNAs, address these limitations by protecting miRNAs from degradation and enabling their precise targeting. As such, harnessing EVs for miRNA delivery has the potential to redefine therapeutic approaches and offer innovative strategies to tackle the most challenging diseases of the 21st century. In this perspective, we examine the advantages and hurdles of EV-mediated miRNA delivery, from state-of-the-art research to its path toward commercialization. This article aims to inspire readers with the promise of EVs and miRNAs as revolutionary tools for future medicine.

## 1. Introduction

From the discovery of penicillin to the development of mRNA vaccines, the Nobel Prize in Medicine has highlighted advancements that reshape healthcare, driving clinical innovations that directly impact patient care. This year’s award continues this tradition, honoring pioneering work that has deepened our understanding of miRNAs and their pivotal regulatory roles in gene expression. Until the 1990s, gene regulation was largely limited to the transcription of DNA into mRNA and its translation into proteins. Yet, despite this understanding, a pivotal question persisted: how does the same DNA blueprint give rise to the multitude of distinct cells and tissues that comprise the human body?

This question captivated this year’s laureates, Victor Ambros and Gary Ruvkun. During their postdoctoral research in Robert Horvitz’s laboratory, they investigated the development of *C. elegans*, a nematode widely used as a model organism for understanding cellular differentiation. In examining the two mutants, they discovered that the lin-4 gene negatively regulates lin-14. ^1^ Later, upon establishing their own labs, Ambros discovered that lin-4 produced a short, non-coding RNA sequence (now widely known as microRNA) while Ruvkun, working independently, found that this sequence inhibited lin-14 by binding to complementary regions in its mRNA. ^2^ These two parallel discoveries laid the groundwork for the field of miRNA research. Initially believed to be unique to *C. elegans*, the discovery by the Ruvkun lab of another miRNA encoded by the *let-7* gene, a gene that is highly conserved across species, paved the way for understanding miRNA-mediated regulation as a fundamental biological process across organisms. ^3^ This groundbreaking finding redefined the role of RNAs beyond protein synthesis and culminated in the 2024 Nobel Prize in Physiology or Medicine being awarded to Ambros and Ruvkun. ^3^

**Fig. 1.**
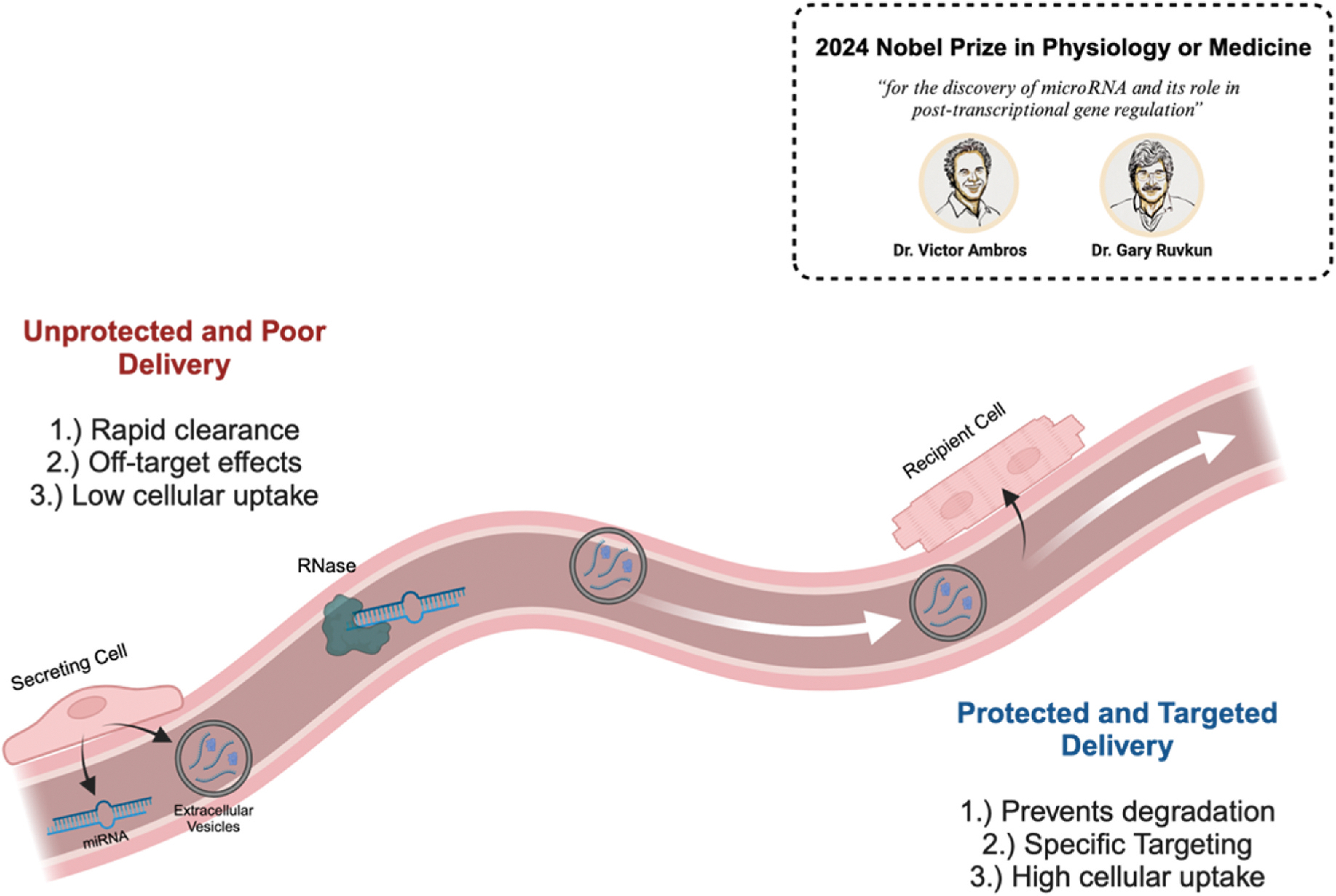
Encapsulating miRNA in extracellular vesicles for therapeutics. EV-based miRNA therapies represent a promising frontier in precision medicine, offering targeted delivery and protection of miRNA within the circulatory system.

The implications of miroRNAs (miRNAs) extend far beyond their foundational role in gene regulation. They are now recognized not only as central regulators of cellular function but as emerging tools for precise and specialized therapeutic applications. However, delivering miRNAs directly remains a significant challenge due to their instability and susceptibility to degradation in circulation, leading to rapid clearance that diminishes their therapeutic potential. This challenge highlights the need for effective delivery mechanisms to transport miRNAs to target cells. Extracellular vesicles (EVs) as natural carriers of miRNAs offer a promising solution, protecting and guiding these molecules to specific cells while providing a more stable and precise delivery method. Thus, the synergy between miRNAs and EVs presents a promising new frontier in precision medicine (Fig. 1). Together, these technologies hold the potential to transform how we approach drug therapies for the most complex diseases today.

## 2. MicroRNAs: regulators of cellular function and disease

### 2.1. Mechanisms of action of miRNAs

MiRNAs play a crucial role in the post-transcriptional regulation of gene expression by modulating mRNA stability and translation. Understanding this mechanism is crucial for developing miRNA-based therapies, as it forms the basis for their potential therapeutic effects. Their biogenesis begins with the transcription of primary miRNAs (pri-miRNAs) by RNA polymerase II, and their cleavage into precursor miRNAs (pre-miRNAs) in the nucleus. ^4^ Once in the cytoplasm, pre-miRNAs are processed into mature ~22-nucleotide miRNAs and are subsequently loaded within the RNA-induced silencing complex (RISC), guiding it to complementary sequences in target mRNAs, primarily in the 3′ untranslated region (3′ UTR). ^2^ This interaction leads to the repression or degradation of target mRNAs, effectively regulating translation and orchestrating essential cellular functions from development to disease progression.

### 2.2. Importance of disease contexts

MiRNAs play a key role in the development and progression of diseases by regulating important biological processes like cell growth, differentiation, and apoptosis. Their ability to modulate these key processes makes them central to the pathogenesis of various conditions, including cancer, cardiovascular diseases, and neurodegenerative disorders. ^5^ Importantly, this same involvement makes them promising therapeutic targets.

MiRNAs that are overexpressed in cancer cells, known as oncomiRs, are heavily implicated in tumor progression and metastasis. ^6^ Specifically, oncomiRs such as miR-21, let-7, and miR-34 are overexpressed in various cancers, driving pathways that enhance cell proliferation, inflammation, and survival. ^7,8^ Targeting these miRNAs with inhibitors could suppress tumor growth, making them a key therapeutic target of interest. Conversely, restoring tumor-suppressive miRNAs, such as the miR-15a/miR-16–1 cluster frequently lost in cancers due to genomic fragility, offers a promising strategy to activate apoptotic and growth-regulatory pathways. ^9^ For example, therapeutic modulation of miR-19a and miR-19b, which activate the PI3K/AKT pathway, could inhibit the survival of cancer cells. ^10^

In contrast to oncomiRs, the repertoire of cardiac-specific miRNAs, known as myoMiRs, is considerably smaller yet equally significant. These miRNAs are central to maintaining cardiac health and their dysregulation has been linked to an array of cardiac pathologies. For example, miR-133, one of the most abundant miRNAs in the heart, is downregulated following acute myocardial infarction (MI). Similarly, miR-1, also reduced following MI, is associated with detrimental cardiac remodeling. ^11^ Both miRNAs are involved in the pathogenesis of cardiac hypertrophy, where their downregulation contributes to gene re-expression linked to electrical remodeling. ^12^ As such, targeted therapy has the potential to reverse these effects to attenuate detrimental injuries to the heart. ^13^

Neurodegenerative diseases further highlight the diverse roles of miRNAs across organ systems. In Alzheimer’s disease, the loss of miRNAs such as miR-9 and miR-125b is linked to neuroinflammation and impaired neuronal survival. ^14^ In Parkinson’s disease, miRNAs regulate alpha-synuclein, a protein central to the disease’s pathogenesis. ^15^ Modulating these miRNAs could reduce oxidative stress and neuronal cell death, opening new avenues for therapeutic intervention. Due to their ability to regulate multiple genes and pathways simultaneously, miRNAs hold significant promise for treating complex diseases. Their network-level influence makes them especially valuable for addressing pathologies with intricate, multifaceted mechanisms.

### 2.3. Challenges in developing miRNAs as drugs

Given the dysregulation of key miRNAs across various diseases, their implication in disease has garnered significant attention in recent research. However, ensuring miRNA stability in the bloodstream is a significant challenge due to rapid degradation by RNases, which drastically limits their bioavailability and therapeutic potential. Chemical modifications, such as 2′-*O*-methylation, 2′-fluoro-modifications, and the incorporation of locked nucleic acids (LNAs), can enhance miRNA stability by reducing susceptibility to nucleases. ^16^ However, these modifications may disrupt miRNA binding affinity or interfere with cellular uptake, potentially diminishing therapeutic efficacy. To balance miRNA stability and bioactivity, researchers have optimized chemical modifications by selectively altering non-essential nucleotides to maintain target interaction while enhancing stability. ^17^ For example, incorporation of 2′-F and 2′-Ome ribose modifications demonstrated improved stability. ^18^ Additionally, encapsulation within carriers such as nanoparticles has been explored to mitigate the need for chemical modification. Of which, EVs present a particularly promising solution. ^19^ As natural carriers of miRNAs, EVs not only protect them from degradation but also facilitate targeted delivery to specific cell populations. By leveraging the intrinsic properties of EVs, this strategy could significantly enhance the therapeutic potential of miRNAs.

## 3. Extracellular vesicles: natural carriers of MicroRNAs

### 3.1. Overview of EVs and their types

EVs are lipid-bilayer-enclosed particles naturally secreted by cells, playing a vital role in intercellular communication, and are natural biological carriers. They transport molecular cargo, including miRNAs, to recipient cells, thereby influencing various physiological and pathological processes. ^20^ They are categorized into exosomes (30–150 nm), which form from multivesicular body fusion and are ideal for miRNA delivery, microvesicles (100–1000 nm), which bud from the plasma membrane in response to stress, and apoptotic bodies (500–2000 nm), released during programmed cell death. ^21^ Recent studies have identified new subclasses of EVs such as oncosomes and migrasomes, which differ in their biogenesis, size, and function, further enhancing our understanding of these complex structures. ^21^

### 3.2. Mechanisms of EV-mediated delivery of miRNAs

#### 3.2.1. MiRNA sorting into EVs

Cells have evolved specific mechanisms for selectively loading and targeting miRNAs to recipient cells via EVs. The endosomal sorting complex required for transport (ESCRT) pathways plays a critical role in this process, facilitating the formation of multivesicular bodies where miRNAs are loaded before EV release. Within this pathway, ESCRT-II forms filaments that promote membrane bending and sever the exosomal neck from the membrane. ^22^ In addition to ESCRT-dependent pathways, there are also ESCRT-independent mechanisms that sort miRNAs based on specific motifs or associated proteins. For example, the hnRNPA2B1 motif governs the loading of specific miRNA subsets into exosomes, while Y-box 1 binds to miRNAs for sorting, resulting in EVs enriched in proteins that regulate cell sorting, biogenesis, and targeting. ^23,24^

#### 3.2.2. Protection of miRNAs from degradation

The structural attributes of EVs play a pivotal role in stabilizing miRNAs during transport, shielding them from the dynamic extracellular environment. The phospholipid bilayer of EVs acts as a robust physical barrier, preventing degradation by RNases that threaten naked miRNAs in circulation. ^20^ Enriched with sphingolipids, cholesterol, and tetraspanins, the EV membrane enhances stability, protecting miRNAs from oxidative stress, immune detection, and premature degradation. ^25^ Once taken up, miRNAs are stored within multivesicular bodies before being directly release into target cells, bypassing circulation and minimizing degradation risks. ^26^ Additionally, EV-associated proteins such as Argonaute 2 (AGO2) and Y box protein 1 (YB1) selectively stabilize miRNAs, further enhancing their longevity. ^27,28^ As a result, miRNAs remain intact as they traverse extracellular spaces, persisting for hours or even days. ^29^ This protective effect, conferred by EV membrane composition and protein interactions, allows EVs to navigate bodily fluids undetected by the immune system while preserving miRNA bioavailability. These advantages position EVs as promising vehicles for miRNA-based therapies. ^30^

#### 3.2.3. Targeting specific cells and tissues

Beyond protecting miRNAs from degradation, EVs are equipped with surface proteins and lipids, such as integrins (e.g., αvβ3, αvβ5), tetraspanins (CD9, CD63, CD81), phosphatidylserine, and ceramide, that enable precise targeting to specific cells, including tumors. ^25^ These surface molecules interact with receptors, notably heparan sulfate proteoglycans and lectins, enhancing selective miRNA delivery and initiating signaling pathways that modulate cellular responses. ^31^ Additionally, EVs exhibit tissue-specific tropism, preferentially targeting tissues based on surface markers and signaling molecules. For instance, EVs from mesenchymal stem cells (MSCs) demonstrate a homing ability to inflamed or damaged tissues while endothelial progenitor cell EVs naturally accumulate in ischemic tissues. ^32,33^ This targeted delivery not only enhances the therapeutic potential of miRNAs but also underscores the sophisticated relationship between EV-mediated transport and the modulation of intracellular signaling pathways, ultimately influencing cellular responses and contributing to the broader dynamics of health and disease.

## 4. Therapeutic miRNA delivery using EVs

### 4.1. EVs as natural delivery vehicles

#### 4.1.1. Naturally derived naïve EVs

Cell-based interventions hold great promise for tissue repair and regeneration, with studies demonstrating the efficacy of intravenous injection for brain injuries to cardiosphere transplantation for myocardial infarction. ^34,35^ However, risks of tumorigenesis and immune rejection from cell-therapies result in strict FDA regulations, limiting their use. Furthermore, studies suggest that stem cell secretome can elicit comparable, if not superior, therapeutic responses to those achieved with cell-based therapies. ^36^ In contrast, cell-derived EVs, often termed *naïve EVs* due to their unmodified nature, present a safer, cell-free alternative, delivering miRNAs that similarly regulate cellular functions without the complexities of whole-cell therapies. ^37^

EVs can be sourced from a variety of cell populations, with a growing interest in stem cells and their progeny, including MSCs, embryonic stem cells (ESCs), and induced pluripotent stem cells (iPSCs), each with distinct regenerative and immunomodulatory potential. For example, EVs from iPSC-derived neural stem cells contain miRNAs including miR-320a, miR-103a-3p, and miR-21–5p, which promote neurogenesis. ^38^ Similarly, EVs from iPSC-derived cardiomyocytes are rich in miR-1 and miR-133a, when integrated into a hydrogel patch, significantly reduce infarct size in myocardial injury models. ^39^ MiRNA-enriched EVs from stem cells offer a promising frontier in regenerative medicine, without the assoicated risks of using whole cells, thereby opening doors where traditional cell-based therapies face limitations.

#### 4.1.2. Engineered EVs for inhibition or mimicry

While naturally derived EVs offer safe, biologically compatible options for delivering miRNAs, engineering these vesicles further enhances their therapeutic precision, specifically, by allowing for disease-specific loading of miRNAs and targeting of EVs. With surface modifications, such as cardiac homing peptides, EVs can also achieve targeted delivery, as demonstrated in myocardial infarction models. ^40,41^ In addition, unlike synthetic nanoparticles, EVs navigate biological barriers and evade immune detection more effectively, allowing for greater therapeutic performance. ^42,43^

A diverse array of loading techniques is advancing the use of EVs as carriers for therapeutic miRNAs, facilitating targeted gene silencing or activation through miRNA inhibitors and mimics. ^44^ Passive loading methods, such as incubation and freeze-thaw cycles, offer simplicity, as illustrated by studies using EVs to deliver paclitaxel for prostate cancer treatment. ^45^ However, it has a low loading efficiency of ≤5 %. ^46^ In contrast, active methods, including electroporation and sonication, induce temporary membrane pores, allowing for efficient miRNA incorporation. ^47^ Loading efficiency using electroporation is typically <10 % though some optimized protocols have yielded higher efficiencies at 30 %. ^48^ To achieve sustained therapeutic effects, parent cells can be genetically modified to produce miRNA-enriched EVs. For example, MSC-derived EVs overexpressing miRNA-let-7c have shown significant efficacy in reducing renal fibrosis. ^49^ Notably, transfection yields high loading efficacies at over 50 %. ^50^ While these methods have demonstrated significant functional effects upon delivery, continued advancements in loading techniques and efficacy are essential to fully realize the therapeutic potential of EV-based miRNA delivery.

**Fig. 2.**
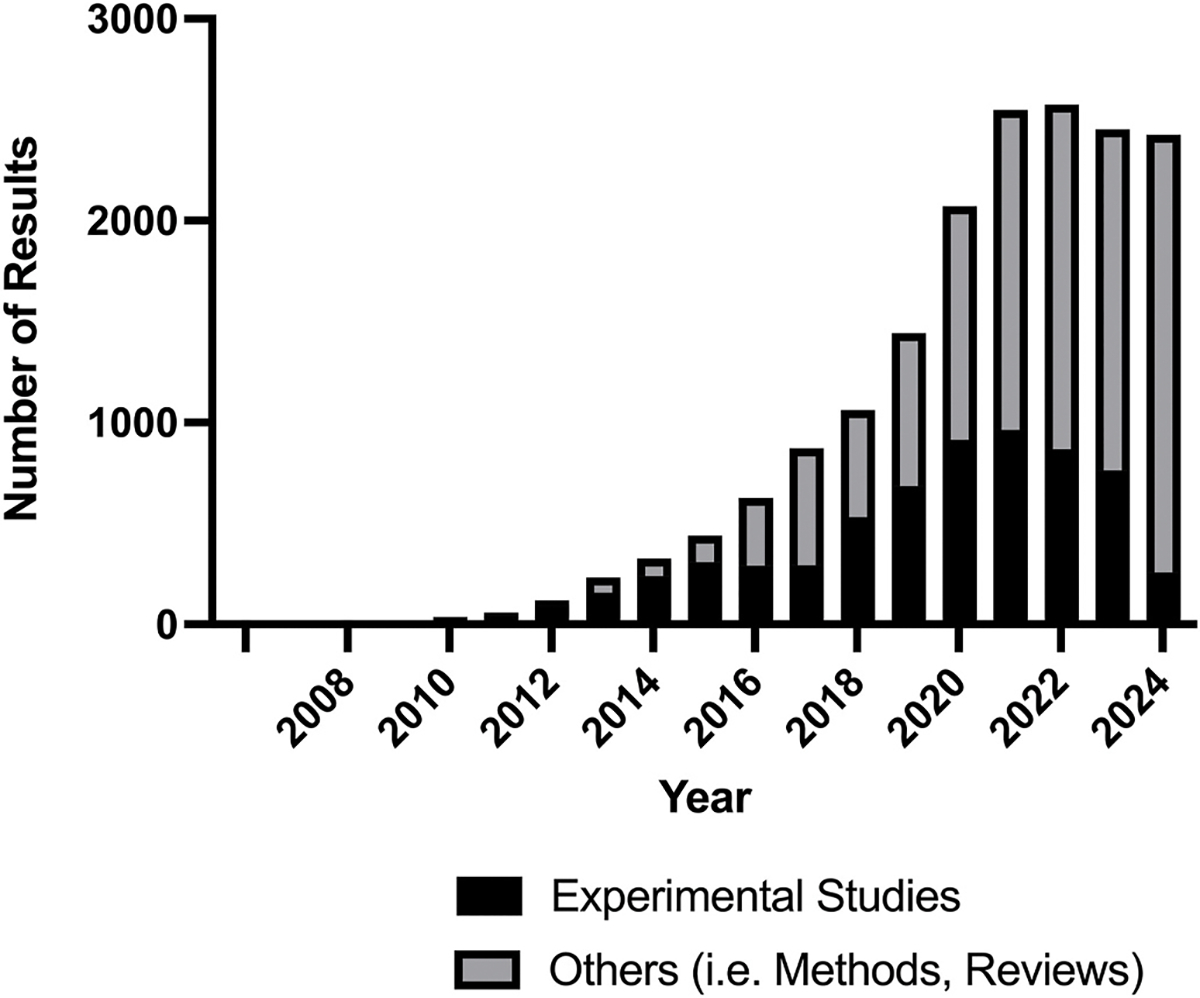
Growth of miRNA and extracellular vesicle research (2006–2024). Number of mentions related to miRNA and extracellular vesicles as shown on PubMed from 2006 to 2024 illustrates an exponential increase in experimental studies and related literature over the years.

Together, these advances in EV engineering, from optimized loading methods to parent-cell modifications, are expanding the therapeutic potential of EVs across various disease models. Literature searches on EVs and miRNAs have increased significantly over the past 15 years, encompassing both experimental studies and broader scientific discussions (Fig. 2). Although clinical studies remain limited, a similar upward trend is evident, highlighting the growing interest in their translational potential.

### 4.2. Limitations of EV-based delivery systems

Despite their potential to enhance miRNA stability, EV-based delivery systems present significant challenges that must be addressed for clinical translation. The inherent heterogeneity of EV populations, variations in size, molecular composition, and functional properties, can result in inconsistent therapeutic outcomes, underscoring the need for refined isolation and characterization protocols. ^51^ Advanced analytical techniques such as high-resolution flow cytometry and mass spectrometry enable precise identification of EV subpopulations and cargo profiling. Imaging flow cytometry differentiates EV subsets by size and surface markers, while mass spectrometry-based proteomics and lipidomics provide comprehensive molecular characterization, offering critical insights to mitigate EV heterogeneity. ^52,53^

To translate these analytical advancements into practical applications, optimizing separation techniques is imperative for enhancing the reproducibility and reliability of EV-based therapies. Isolation strategies such as size-exclusion chromatography (SEC), asymmetricflow field-flow fractionation (AF4), and tangential flow filtration (TFF) have demonstrated efficacy in improving purity and scalability while reducing batch-to-batch variability. ^54^ Additionally, the standardization of production methods, including ultracentrifugation and precipitation, is essential to ensuring regulatory compliance and facilitating large-scale manufacturing. ^55,56^

Establishing stringent quality analysis and control metrics is fundamental for the clinical implementation of EV-based therapeutics. While tetraspanins serve as conventional markers for benchmarking EVs, additional parameters such as particle-to-protein ratio, RNA content, zeta potential, and rigidity provide further measures to enhance reproducibility. ^57^ Furthermore, maintaining high-quality cell cultures, ensuring the purity of isolated EVs, standardizing isolation methods, and evaluating the safety of packaging are critical quality control measures. ^58^ Recent efforts to implement quality control into EV engineering have also leveraged MSC morphology, specifically, analyzing cell shape to predict the anti-inflammatory activity of MSC-derived EVs. ^59^ Incorporating these rigorous quality assessments will be critical for ensuring the consistency, safety, and regulatory approval of EV-based therapies. ^60^

## 5. Commercialization of miRNA-based EV therapies

### 5.1. Promising preclinical and clinical trials

Recent clinical trials have investigated EV-based miRNA therapies as novel therapeutic platforms for treating complex diseases. ^60^ By leveraging the natural targeting abilities and adaptable properties of EVs, researchers hope to address unmet needs in precision medicine where conventional therapies often fall short. While many miRNA therapies in trials currently rely on liposomes or direct injection, EV-based trials often focus on delivering larger molecules such as proteins and drugs, and only a limited number of exosomal miRNA therapies have advanced into clinical trials, with few reaching Phase 3. ^61,62^

In 2014, a Phase 2/3 clinical trial was conducted using umbilical cord-derived MSC EVs to treat chronic kidney disease (CKD). Forty patients with stage III and IV CKD received either EVs or a placebo, and kidney function, along with immune activity, was monitored. Those treated with MSC EVs showed improved glomerular filtration rates and increased plasma levels of TGF-Beta and IL-10, demonstrating the therapeutic potential of natural miRNA-containing EVs. ^63^ Another Phase 1 trial investigated grape-derived exosomes enriched with miR-169 to reduce oral mucositis in cancer patients undergoing chemotherapy and radiation. Daily administration of these EVs exhibited significant anti-inflammatory effects, highlighting the promise of plant-derived EVs in therapy. ^64^ Additionally, an ongoing Phase 3 trial is evaluating bone marrow MSC-derived EVs for Acute Respiratory Distress Syndrome (ARDS). Patients are orally administered EVs over 60 days, with outcomes such as survival time and ventilator-free days being assessed. Results, expected in early 2025, may further validate MSC-derived EVs for ARDS treatment. ^65^

It is noteworthy, however, that several EV-based miRNA therapies have been terminated, withdrawn, or currently have unknown statuses. ^66^ While some of these challenges stem from safety and efficacy concerns, many are attributed to factors such as insufficiently eligible participants, company bankruptcies, or shifts in project priorities. ^67,68^ As extracellular vesicle research remains an emerging field, its early-stage development presents challenges in subject recruitment and funding acquisition. Therefore, as the field progresses, increasing awareness among both the scientific community and potential investors is essential to fostering support for this transformative technology. The limited success of these trials highlights the need for continued research and development to overcome existing hurdles, improve the effectiveness of EV-based miRNA therapies, and advocate for greater support in extracellular vesicle research.

### 5.2. Current commercial and therapeutic efforts

The intersection of miRNAs and EVs has garnered significant attention in recent years, with more than 10,000 publications mentioning both terms in their titles and 25 ongoing clinical trials. ^69,70^ The exosome market is projected to expand at a compound annual growth rate of 28.38 % from 2024 to 2030, while the miRNA market is currently valued at $12.87 billion. ^71,72^ Despite this burgeoning interest, no FDA-approved commercial product utilizing miRNA-based EV drug delivery has yet emerged. To improve the success rate of translating EV research into the market, advancements in both clinical trial design and EV technology are essential. For instance, stratifying patients based on biomarker profiles could help identify those most likely to benefit from miRNA-based EV therapy. ^73^ Additionally, refining EV engineering, such as optimizing miRNA loading, enhancing targeted delivery, and improving large-scale manufacturing, can increase therapeutic precision and consistency, ultimately facilitating clinical translation and commercialization.

Table 1 summarizes commercial efforts to deliver both naïve and EV-based miRNAs for therapeutic applications across various disease areas. Across the market, there is a strong focus on developing highly efficient purification methods, enhancing tissue-specific targeting, and creating customizable EV platforms for payload loading, some of which are highlighted in Table 1.

### 5.3. Safety and efficacy concerns

#### 5.3.1. Off-target effects and unintended consequences

While EV-based miRNA therapies hold great potential, challenges such as off-target effects and immune responses continue to impede their clinical development. As miRNAs can influence the expression of numerous genes, these therapies carry the risk of off-target effects, which may lead to unintended gene regulation of critical cellular processes. To mitigate these effects, researchers are exploring methods to engineer EVs with specific targeting receptors to direct them toward the desired cell or tissues. ^40^ However, achieving reliable targeting remains challenging, partly due to the complex interplay between EV surface proteins and cell receptors, which is not yet fully understood. This knowledge gap also impacts the body’s immune responses to EVs, as some studies have shown that EVs may provoke inflammation depending on their origin and delivery method. ^74^ Therefore, continued efforts to explore the surface protein on EVs could help minimize both off-target effects and immune activation, thereby advancing the safety and efficacy of exosomal miRNA therapies. Furthermore, refining experimental models to better mimic human physiological conditions such as utilizing organoid systems and humanized animal models could provide deeper insights into EV biodistribution, unintended gene interactions, and immune responses, ultimately leading to more precise therapeutic design and reducing off-target effects in clinical applications.

#### 5.3.2. Regulatory hurdles and approval processes

The complex nature of EVs presents significant regulatory challenges, as agencies like the FDA and EMA work to classify EV-based miRNA therapies within existing frameworks. Despite these hurdles, exosomes are expected to have a smoother approval process than cellular therapies due to their simpler composition and reduced risk of immune rejection. ^75,76^ In 2019, the FDA announced exosomes used in human disease treatment to be classified as drugs and biological products. ^77^ However, the lack of standardized quality control measures for EV production continues to hinder the progression of clinical trials and their translation beyond laboratories. In January 2023, the FDA initiated efforts to establish specific control parameters for MSC-derived EVs. Nevertheless, extensive characterization will still be required for EVs derived from other cell types as they move toward clinical approval. ^59^ Establishing clear regulatory guidelines and standardized protocols will also be critical to ensure the consistency, safety, and efficacy of EV-based therapies, ultimately facilitating smoother clinical trial design and accelerating their path to market. Early regulatory progress by companies such as ExoPharm and Evox Therapeutics underscores the potential of EV-based therapeutics and offers a promising outlook for their clinical application.

### 5.4. Intellectual property and market considerations

Securing patents for miRNA-based drugs presents a unique challenge, as naturally occurring sequences are often not patentable. ^78^ To address this, companies are exploring patents for modified miRNAs, engineered EVs, and innovative delivery methods, which could offer the necessary protection needed in this rapidly expanding field. The rise of EV-based miRNA therapeutics, especially in areas like cancer, cardiovascular diseases and neurodegenerative diseases, has attracted intense interest, with numerous companies racing to develop viable therapies. However, producing EVs at a commercial scale remains costly, partly due to stringent quality control and storage requirements. Current research efforts are striving to optimize large-scale EV production to make these therapies more accessible and cost-effective. ^79^ These intellectual property and manufacturing challenges need to be considered in the design of clinical trials, where strategic patient selection, biomarker-driven enrollment, and optimized dosing regimens can help demonstrate clear therapeutic benefits, strengthening the case for regulatory approval and commercial viability. With substantial investments fueling advances in EV-based miRNA therapies, these efforts hold promise for transforming treatment options in some of the most challenging disease areas. ^80^

## 6. Conclusion and future perspectives

The Nobel Prize’s recognition of miRNAs highlights their significant impact on basic biological science and paves the way for innovative miRNA-based therapies that could reshape modern medicine. However, fully realizing the potential of EV-based miRNA therapy will require overcoming key obstacles: minimizing off-target effects, ensuring stability in circulation, and developing scalable production methods. Furthermore, since most miRNAs naturally exist in a freeform state within the human body, it is essential to evaluate their individual therapeutic roles and determine whether they can be effectively delivered via EVs. Addressing these challenges will necessitate interdisciplinary collaboration across scientific, regulatory, clinical, and intellectual property fields.

Building on recent advancements, multifunctional delivery systems enable the co-delivery of EVs, proteins, and therapeutic agents like platelets. Platelets, which carry bioactive molecules, promote cardiac repair both independently and within biomaterial scaffolds. ^81,82^ Their co-delivery with cells has shown significant therapeutic potential, and integrating EVs into these combined strategies could enhance therapeutic efficacy for complex diseases. Hydrogels offer a promising platform for controlled co-delivery of EVs and other therapies. While widely used in cell therapy, their potential for EV delivery remains underexplored. ^83,84^ Co-encapsulating EVs with other therapeutics in hydrogels could enhances tissue retention and enable controlled release. ^85^ Alternatively, co-encapsulation in nanoparticles provides another viable strategy for delivering multiple agents simultaneously.

Engineered EVs are also a promising avenue being explored for drug and protein co-delivery. For example, EV-mediated delivery of TRAIL and DINA has been investigated for tumor-targeted therapy, ^86^ while loading EVs with 5-FU and a miR-21 inhibitor has shown potential in reversing drug resistance. ^87^ Continued exploration of engineered EVs for their combined application with other therapeutic modalities could provide a powerful multimodal strategy to address complex 21st-century diseases that lack a singular target. To assess co-delivery efficacy, it is essential to compare the therapeutic impact of each component individually and in combination, both in vitro and in vivo. Organ-on-a-chip technology offers a valuable platform to decouple individual contributions while facilitating the evaluation of their combined effects across different cell populations in a controlled manner. ^88^ Additionally, evaluating biosafety, delivery efficiency, and other key variables is crucial for optimizing these integrated therapeutic strategies.

EV-based miRNA therapies also offer a promising approach to advancing personalized medicine by customizing EVs to deliver miRNA profiles tailored to individual patients. This approach has the potential to address interpatient variability, ensuring a more precise and effective treatment for each individual. While the full potential of EVs in precision medicine is still being explored, these advancements signal the emergence of a new era in individualized therapies that are closely aligned with each patient’s unique biological profile. By leveraging their natural ability to selectively package and deliver therapeutic cargo, EVs provide a powerful platform for precision medicine, offering a uniquely adaptable strategy for tailoring treatments to diverse disease presentations and patient needs. Furthermore, leveraging the co-delivery of EVs with other therapeutic agents will enhance patient-specific treatment by optimizing the release profile and therapeutic composition, ultimately improving individual outcomes.

As we celebrate this year’s Nobel Prize in Medicine, we look forward to harnessing the full potential of miRNAs in improving human health and well-being globally. Further exploration into the design, delivery, and regulation of EV-based miRNA therapies has the potential to lead to impactful clinical applications, transforming traditional drug therapies into more personalized, targeted approaches for complex diseases like cancer, cardiovascular disorders, and neurodegenerative conditions. Medicine now stands at a crossroads, shifting from conventional treatments to these more personalized approaches. Now, more than ever, EV-based miRNA therapy emerges as a promising advancement in this endeavor.

## Figures and Tables

**Table 1:** Commercial efforts in miRNA and EV-based miRNA therapy. Summary of commercial activities in miRNA and extracellular vesicle with promising products in clinical stages, with a focus on key companies and products that are leading the commercialization efforts in this field.

Company	Technology	Development Stage	Therapeutic Area	Key Details	Source

Aegle Therapeutics	AGLE-102	Phase 1/2a	Dermatology	Allogenic Bone MSC-derived EVs for burn injury and accelerated regenerative healing	89
Aethlon Medical	Hemopurifier	Preclinical	Oncology, Infectious diseases	Capture tumor-exosomes from several forms of cancer and to clear pathogenic viruses	90
ArunA Biomedical	AB126	Phase 1b/2a	Neurology	Neural-derived exosome that crosses the blood-brain barrier for acute ischemic stroke	91
Avalon Globocare	AVA-201	Preclinical	Oncology	MiR-185 loaded exosomes from MSCs to treat Leucoplakia	92,93
Capricor Therapeutics	CAP-2003	Preclinical	Cardiology	EVs derived from cardiosphere-derived cells, containing miRNAs for inflammation and fibrosis	94
Carmine Therapeutics	REGENT	Preclinical	Oncology	Red blood cell-derived EVs for in vivo gene therapy targeting miR-125	95,96
Direct Biologics	ExoFlo	Phase I/II	COVID-19	EVs from bone marrow MSCs to moderate acute respiratory distress syndrome	97
EnGeneIC	EDV miR-16	Phase 1	Oncology	miR-16 mimic loaded EDV nanocells for targeted cancer therapy	98
Evox Therapeutics	DeliverEx	Preclinical	Rare diseases, Neurology	Load therapeutic cargo into exosomes to target the central nervous system	99
ExoCoBio	RSCE	Preclinical	Cosmetics, Regenerative Medicine	Rose stem cell derived exosome for collagen production of skin fibroblasts	100
ExoPharm	PLEXOVAL	Phase I	Regenerative Medicine, Dermatology	Platelet derived EVs for wound healing treatment	36
Kimera Labs	XoGloPro	IND Approval	COVID-19	Placental, MSC-derived EVs to harvest nature miRNA for treatment of COVID-19 symptoms in adults	101
MiRagen Therapeutics	MRG-110	Phase I	Wound Healing	Inhibition of miR-92a to promote angiogenesis for acute and chronic wound healing	102, 103
MiRagen Therapeutics	MRG-107	Preclinical	Neurology	Inhibition of miR-155 to block neuroinflammation in microglial cells for ALS	94
MiRagen Therapeutics	MRG-201	Phase II	Fibrosis	MiRNA-29 mimic to reduce fibrous scar formation by regulating collagen synthesis	92,93
miRagen Therapeutics	MGN-1374	Preclinical	Cardiology	Inhibition of miR-15 and miR-195 to treat post-myocardial infarction	91
miRagen Therapeutics	MGN-1374	Preclinical	Cardiology	Inhibition of miR-15 and miR-195 to protect against myocardial infarction	104
Organicell Regenerative Medicine	Zofin	Phase I/II	COVID-19	EVs derived from amniotic stem and epithelial cells to target and suppress pro-inflammatory cytokines	105
Regulus Therapeutics	Lademirsen/RG-012	Phase II	Nephrology	Oligonucleotide that binds to and inhibits miR-21 for treatment of Alport syndrome	106
ReNueron	CustomX	Discovery	Oncology	Neural stem cell-derived EV drug delivery platform to deliver miRNAs to specific tissues	102
RION	Purified Exosome Product (PEP)	Preclinical	Regenerative Medicine	Isolate regenerative exosomes from platelets	107
RoosterBio	Stem Cell Exomere Program	Preclinical	Regenerative Medicine	Develop high-grade therapeutic EVs from adult stem cells as a hMSC technology provider	108
Santaris Pharma	Miravirsen	Phase II	Infectious Disease	LNA drug against miR-122 for treatment of hepatitis C virus infection	109
Tavec Pharmaceuticals	TVC-201	Preclinical	Oncology	MiR-195 loaded exosomes for inhibiting the growth of cholangiocarcinoma	110
United Therapeutics	UNEX-42	Phase 1	Pulmonary	EVs from bone marrow-derived MSCs for bronchopulmonary dysplasia in preterm infants	111
